# Case Report: Postoperative ascites: allergic reaction to the drainage tube in a 12-year-old patient

**DOI:** 10.3389/fsurg.2024.1409673

**Published:** 2024-10-30

**Authors:** Ren-sen Jiang, Jing Lao, Huan-sheng Wang, Miao-bing Wu, Bing Wang, Jian-yao Wang

**Affiliations:** ^1^ Shenzhen Pediatrics Institute of Shantou University Medical College, Shenzhen, Guangdong, China; ^2^ Department of General Surgery, Shenzhen Children's Hospital, Shenzhen, Guangdong, China; ^3^ Shenzhen Children's Hospital of China Medical University, Shenzhen, Guangdong, China

**Keywords:** allergy and immunology, ascites, suction drainage, silicone, mucinous cystadenoma, case report

## Abstract

Allergic reaction to the silicone is rare in children and as a result very little experience has been reported on symptom and treatment. We presented a case involving a child who experienced prolonged ascites following a surgery of placing an abdominal drainage tube, characterized by the ongoing drainage of clear, light-yellow fluid at a rate of 250 mL/day through the drainage tube for 36 days. Examination of the ascitic fluid revealed an abnormal elevation in eosinophil proportion, which exhibited positive response to anti-allergic treatment. Subsequent to the removal of the drainage tube, the ascites gradually resolved. In conclusion, we presented here the first and youngest case of allergic ascites associated with drainage tube after surgery of ovarian mucinous cystadenoma, it is imperative not to overlook the possibility of drainage tube allergy in the diagnostic process.

## Introduction

Ovarian mucinous cystadenoma is a common benign tumor in children (1). The etiology of postoperative ascites is multifaceted, primarily associated with factors such as infection, damage to lymphatic vessels, the extent of surgical trauma, as well as potential injuries to the ureter and intestine (2). Prolonged ascites increases the risk of complications such as spontaneous peritonitis, hypoalbuminemia, hyponatremia, and dehydration, greatly prolonged the time of hospitalization (3, 4). Reports of ascites cases related to silicone drainage tube allergies are rare, and there are no standard guidelines for the optimal treatment of ascites related to drainage tube allergies. Herein, we present a clinically confirmed patient with postoperative ascites associated with silicone drainage tube allergy.

## Case description

**Figure 1:**
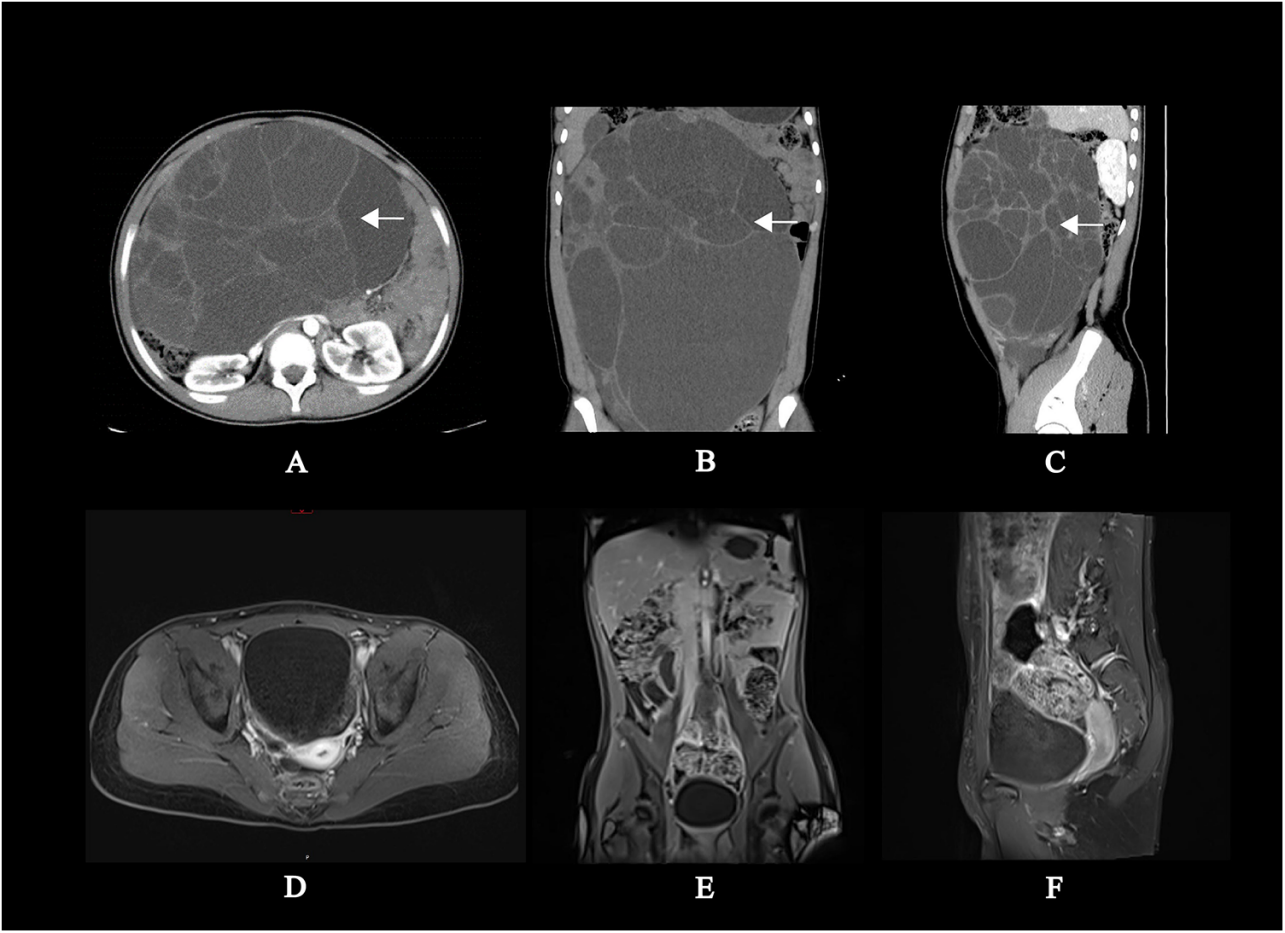
Preoperative CT scan contrast with postoperative MRI scan of abdomen. **(A–C)** Presents plain and enhanced CT scan of preoperative abdomen: There was a colossal divided cystic mass with clear boundaries about 23 cm **×** 15 cm **×** 32 cm (left and right × front and rear × up and down) in the abdominal and pelvic cavities. **(D–F)** Presents plain and enhanced MR scan of postoperative abdomen: There was multiple free fluid signal shadows in the abdominal and pelvic cavities; Local continuity interruption on the right abdominal wall, with a drainage tube visible inside. The size and morphology of the right ovary was in the normal range contrast with left ovary. No lymph node enlargement was observed in the abdominal cavity or retroperitoneum. No abnormal signals were observed in various organs, bones, and surrounding soft tissues.

**Figure 2:**
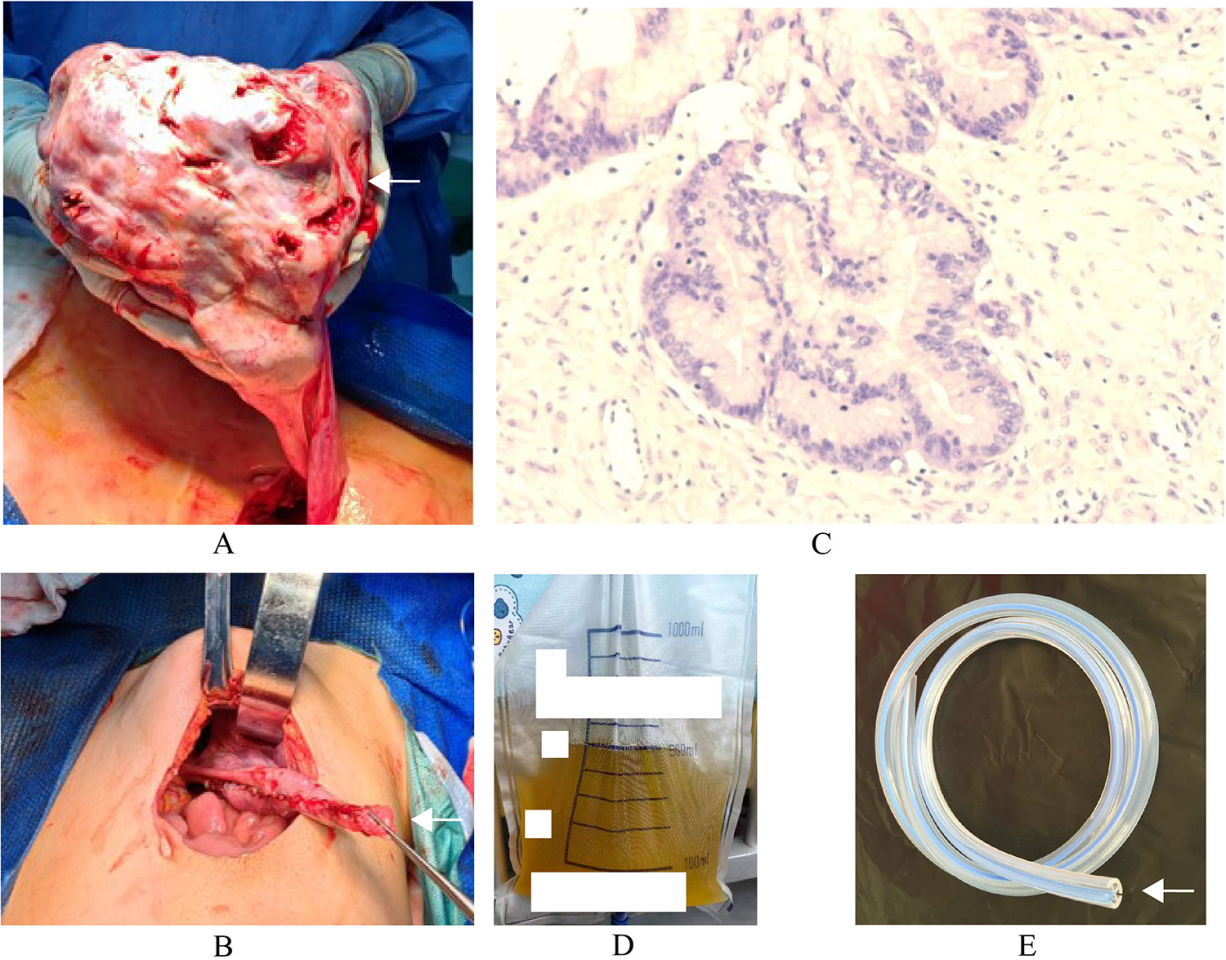
The surgical findings and pathology of neoplasm. **(A)** The subcapsular cyst about 30 cm × 28 cm × 15 cm originates from the right ovary. **(B)** The ovarian cortex was sutured to a size of approximately 4 cm × 3 cm × 2 cm without active bleeding. **(C)** Pathology of neoplasm: The tumor presents as a multicystic structure, lined with a single layer of columnar epithelium, with focal papillary hyperplasia. The epithelium shows pseudostratified hyperplasia, while the small lesion epithelium shows mild atypical hyperplasia. **(D)** The light-yellow clear ascites. **(E)** The silicone drainage tube.

**Figure 3:**
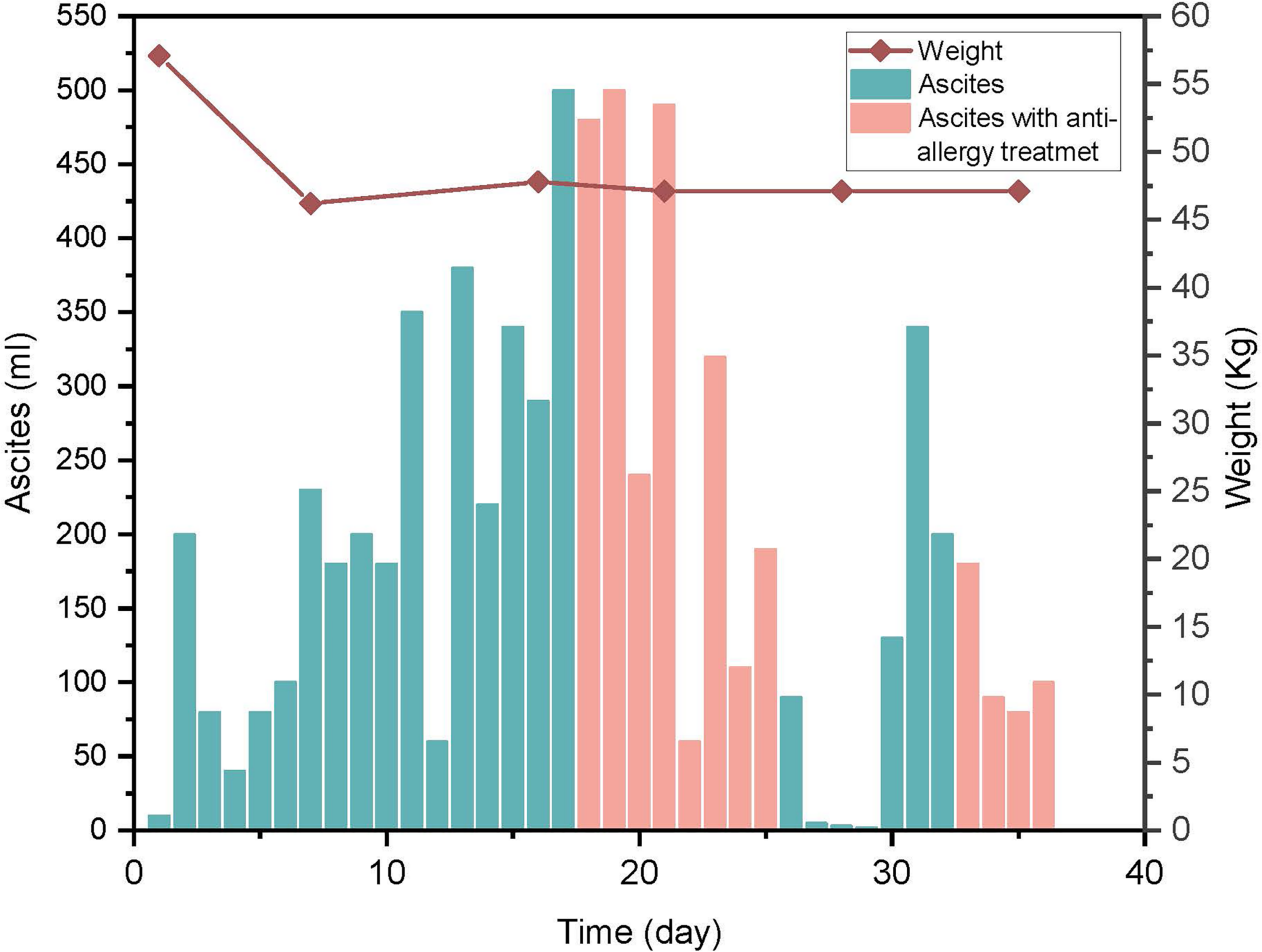
Variation of ascites and body weight. Time represents days post-surgery. The column graph represents the volume of ascites, and the line graph represents the changes in body weight. The column graph in pink color represents the ascites during anti-allergy treatment. Ascites significantly decreased with anti-allergic treatment, and after discontinuing anti-allergic treatment, ascites significantly increased after 4 days.

A 12-year-old female patient was hospitalized due to “abdominal distension for more than 2 days”. A CT scan revealed a substantial abdominal mass (Figures 1A–C) closely associated with the ovary. Due to the large size of the tumor, in order to prevent the tumor from growing further and causing more serious compression symptoms, based on the results of CT scan, the ovarian cystadenoma was diagnosed, and the boundary of tumor was clear, and surgical removal was feasible, her family accepted surgery after informing the parents of the details and risks of the operation. Intraoperatively, a cystic-solid mass originating from the right ovary, measuring approximately 30 cm × 28 cm × 15 cm, with septations in the cyst wall was identified (Figure 2A). The cyst fluid, turbid and viscous, resembled gelatin. Approximately 5,000 mL of viscous fluid was aspirated, and complete removal of the ovarian tumor was performed, preserving and reconstructing the residual ovary to dimensions of approximately 4 cm × 3 cm × 2 cm (Figure 2B). A peritoneal drainage tube was left in place post-surgery, with no lymph node dissection conducted. Tumor pathology confirmed the diagnosis of ovarian mucinous cystadenoma with epithelial proliferation (Figure 2C). Postoperatively, ascites volume fluctuated continuously at around 250 mL/day. The patient underwent anti-infection treatment, acid and enzyme inhibition, abdominal pressure adjustments, and clamping of the drainage tube. Despite various treatments, abdominal pain persisted, and ascites reduction was not significant. MRI showed ascites without tumor recurrence or implantation (Figures 1D–F), and ultrasound indicated a substantial amount of residual ascites in the abdomen. Further examinations of the ascites revealed an increased eosinophil count (Table 1, Supplementary Tables S1, S2). Suspecting a connection to the silicone drainage tube left in the abdomen, a treatment regimen of loratadine combined with prednisolone acetate was initiated (day 18–day 25). During this period, ascites volume significantly decreased. However, a resurgence occurred after a 4-day cessation of anti-allergic treatment, prompting another course (day 33–day 36) and a gradual reduction in ascites (Figure 3). Notably, drainage fluid volume correlated significantly with anti-allergic treatment, leading to the decision to remove the drainage tube. At follow-up 2 weeks and 1 month after hospital discharge, the patient reported no symptoms such as abdominal pain or discomfort. Ultrasound results showed no evidence of fluid accumulation in the Douglas pouch, signifying the disappearance of ascites symptoms from that point onward.

**Table 1:** Cytological examination of ascites.

Time (day)	Rivalta test	WBC (10^6^/L)	Lymphocytes proportion (%)	Eosinophils proportion (%)
12	++	307	21	58
25	−	182	62	5
32	++	346	20	50
37	−	174	74	8

The character in red indicates abnormal increase in test results and the character in black indicates test result within normal range. Day 25, 37 undergo anti allergic treatment, eosinophils significantly decreased, and Rivalta test turned negative, proving the effectiveness of anti-allergic treatment. It is worth noting that compared to Supplementary Table S1, there is a significant increase in eosinophils in ascites, while there is no abnormality in eosinophils in blood tests.

## Discussion

In the present case, tumor pathology identified the ovarian mass as mucinous cystadenoma with epithelial proliferation, characterized by a lining of single-layer columnar epithelium (Figure 2C). This classification denotes a benign condition with a favorable prognosis (1). While certain mucinous cystadenomas may induce a limited amount of ascites, there is currently no documentation on PubMed regarding ovarian mucinous cystadenoma leading to persistent postoperative ascites.

Medical-grade silicone is a high molecular material synthesized from siloxanes, characterized by outstanding low antigenicity and biocompatibility, making it widely utilized in various medical devices. Although there are currently no reported cases of allergic reactions specifically linked to silicone drainage tubes, several published cases have documented allergic reactions stemming from other medical-grade silicone products. Examples include reactions associated with breast implants (5), continuous positive airway pressure masks (6), toe separators (7), and pacemaker components (8). It's noteworthy that in some instances, allergic reactions to silicone products are suggested to be correlated with additives or by-products in the production process rather than the silicone itself, with substances like tert-butyl alcohol, methyl vinyl ketone, etc. being implicated (9). In this case, it remains uncertain whether the allergen involved is an additive or by-product present in the drainage tube.

In this instance, the patient experienced persistent ascites lasting for 36 days. Extensive testing and experimental interventions were undertaken to rule out potential causes of ascites, including inflammation, tumor recurrence, sudden intra-abdominal pressure fluctuations, lymphatic leakage, and surgical stimulation triggering ovarian secretion. It is worth noting that eosinophilic gastroenteritis and parasitic infections are also important causes of eosinophilic ascites. Eosinophilic gastroenteritis is characterized by eosinophilic infiltration of the gastrointestinal tract, presenting with symptoms such as abdominal pain, nausea, vomiting, and hematochezia (10). These symptoms did not appear in the patient. Gastrointestinal parasitic infections have a well-established correlation with eosinophilic ascites. Parasitic infections often present with abdominal pain. However, no parasites or ova were found in the cytological examination of the ascitic fluid or in the stool microscopy results. Thus, the diagnosis of eosinophilic gastroenteritis or parasitic infection can be excluded.

Subsequently, the identification of an abnormal elevation in eosinophils in the ascetics, along with the success of anti-allergic treatment, led us to pinpoint the silicone drainage tube left in the abdomen as the causative factor. Upon removal of the drainage tube, the patient's ascites resolved without recurrence. Despite the rarity of allergic reactions to silicone drainage tubes, when faced with persist ascites and an abnormal increase in eosinophils in the ascitic fluid, it is imperative not to overlook the possibility of drainage tube allergy in the diagnostic process.

## Conclusion

We propose that in patients with persistent ascites following postoperative drainage tube placement, after eliminating potential contributing factors such as peritoneal inflammation, hypoalbuminemia, abrupt intra-abdominal pressure changes, lymphatic vessel injury, and primary disease factors, the presence of an elevated eosinophil count in the ascitic fluid, even when blood eosinophil levels are within the normal range, may warrant consideration for drainage tube removal. This approach could help mitigate complications like spontaneous peritonitis and hypoalbuminemia, uphold the overall surgical prognosis, and introduce novel diagnostic and therapeutic avenues for managing ascites.

## Data Availability

The original contributions presented in the study are included in the article/Supplementary Material, further inquiries can be directed to the corresponding author.

## Supplementary material

Supplementary Table 1 Venous blood test. “/” represents “not detected”. The postoperative white blood cell count was normal, and the abnormal increase in CRP may be closely related to surgical stimulation. It is speculated that abdominal inflammation is not related to the patient's ascites.

Supplementary Table 2 Liver and kidney function test. The patient had a mild decrease in total protein before surgery and no significant abdominal fluid accumulation; After surgery, there was a slight decrease in protein and it remained basically unchanged, which was not significantly related to changes in ascites. It is speculated that hypoalbuminemia is not related to the patient's abdominal fluid accumulation.
