# Supplementary figures and images for: Case Report: Postoperative ascites: allergic reaction to the drainage tube in a 12-year-old patient
Source: Front Surg. 2024 Oct 30;11:1409673. doi: 10.3389/fsurg.2024.1409673 (PMC11557521; doi:10.3389/fsurg.2024.1409673)

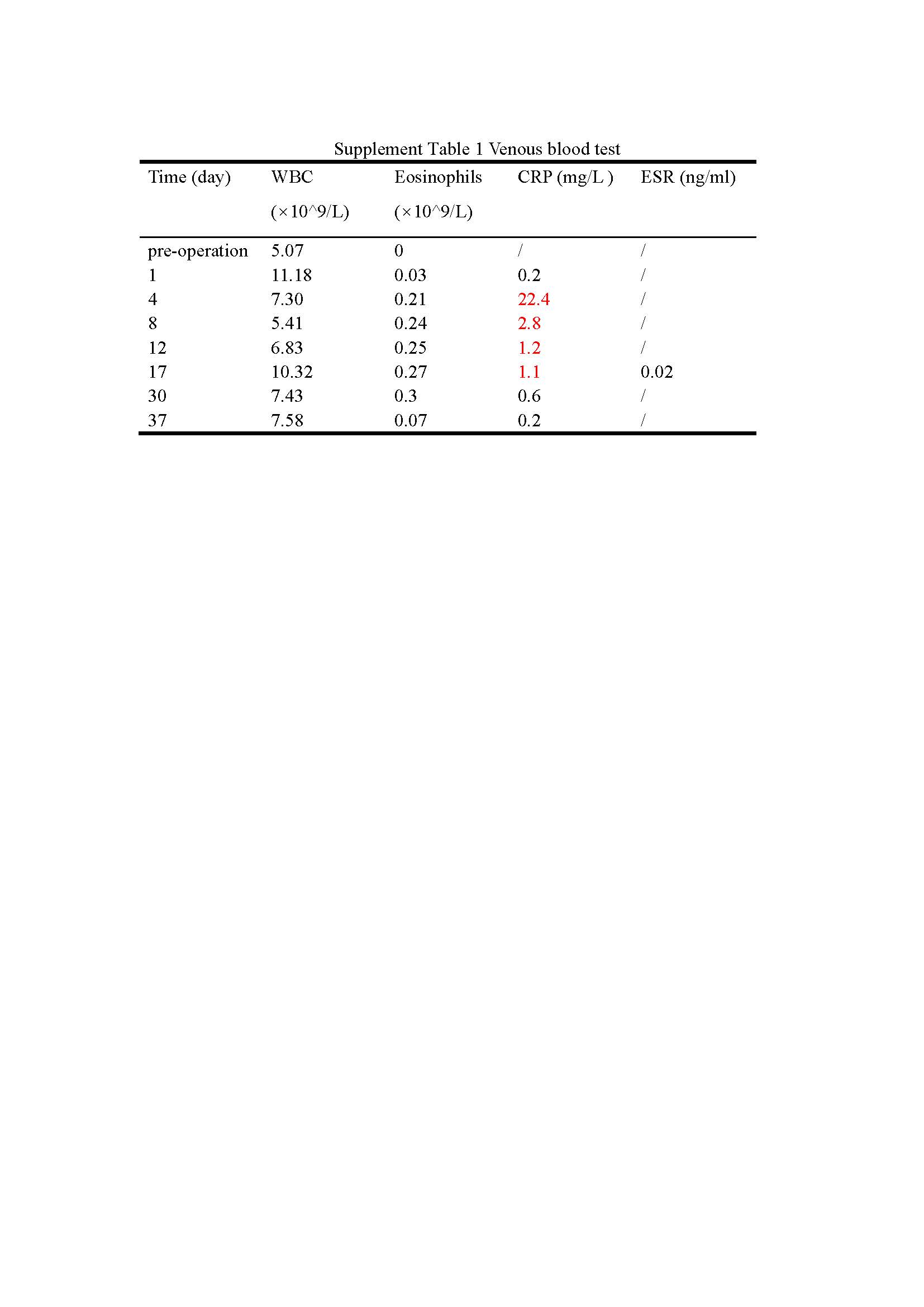

Supplement: Supplementary Table 1 — Venous blood test. “/” represents “not detected”. The postoperative white blood cell count was normal, and the abnormal increase in CRP may be closely related to surgical stimulation. It is speculated that abdominal inflammation is not related to the patient's ascites. [file Image1.jpeg]

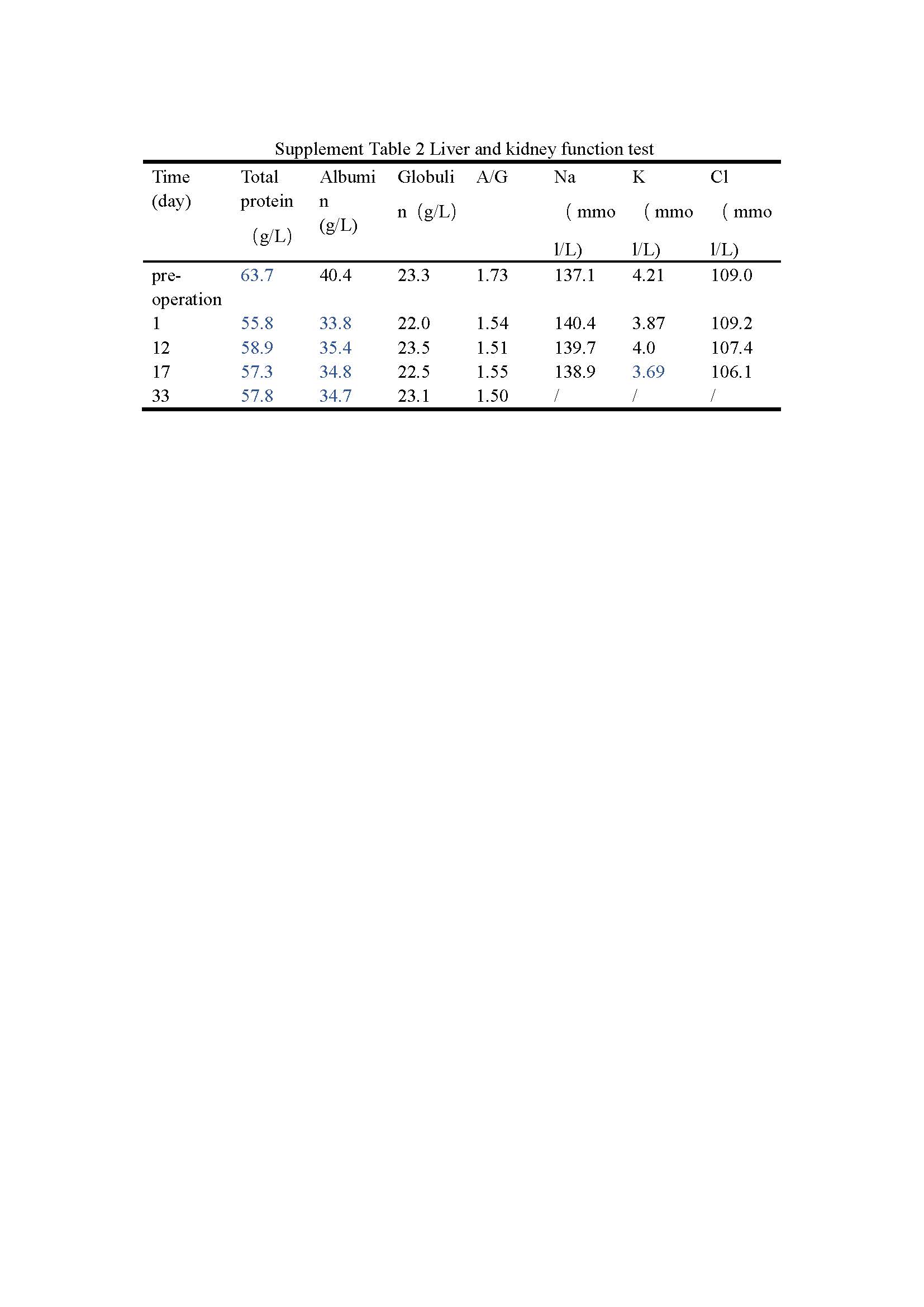

Supplement: Supplementary Table 2 — Liver and kidney function test. The patient had a mild decrease in total protein before surgery and no significant abdominal fluid accumulation; After surgery, there was a slight decrease in protein and it remained basically unchanged, which was not significantly related to changes in ascites. It is speculated that hypoalbuminemia is not related to the patient's abdominal fluid accumulation. [file Image2.jpeg]
